# Biceps Brachii Muscle Synergy and Target Reaching in a Virtual Environment

**DOI:** 10.3389/fnbot.2019.00100

**Published:** 2019-12-10

**Authors:** Liang He, Pierre A. Mathieu

**Affiliations:** Department of Pharmacology and Physiology, Biomedical Engineering Institute, Université de Montréal, Montréal, QC, Canada

**Keywords:** biceps brachii, muscle synergy, upper limb posture classification, target reaching, virtual cube, myoelectric prosthesis

## Abstract

A muscular synergy is a theory suggesting that the central nervous system uses few commands to activate a group of muscles to produce a given movement. Here, we investigate how a muscle synergy extracted from a single muscle can be at the origin of different signals which could facilitate the control of modern upper limb myoelectric prostheses with many degrees of freedom. Five pairs of surface electrodes were positioned across the biceps of 12 normal subjects and electromyographic (EMG) signals were collected while their upper limbs were in eight different static postures. Those signals were used to move, within a virtual cube, a small red sphere toward different targets. With three muscular synergies extracted from the five EMG signals, a classifier was trained to identify which synergy pattern was associated with a given static posture. Later, when a posture was recognized, the result was a displacement of a red sphere toward a corner of a virtual cube presented on a computer screen. The axes of the cube were assigned to the shoulder, elbow and wrist joint while each of its the corners was associated with a static posture. The goal for subjects was to reach, one at a time, the four targets positioned at different locations and heights in the virtual cube with different sequences of postures. The results of 12 normal subjects indicate that with the muscular synergies of the biceps brachii, it was possible, but not easy for an untrained person, to reach a target on each trial. Thus, as a proof of concept, we show that features of the biceps muscular synergy have the potential to facilitate the control of upper limb myoelectric prostheses. To our knowledge, this has never been shown before.

## Introduction

Important progress has recently been made in the design of multiple degrees of freedom (DoF) upper limb myoelectric prosthesis (Lenzi et al., 2016) and this has led the production of commercially available units, such as the Luke Arm (Mobius Bionics, 2017). Such advanced prostheses can be most valuable to amputees in their daily living. Multiple DoFs implies that multiple control signals have to be derived from EMG signals. To that end, Daley et al. (2012) used a linear discriminant analysis (LDA) to classify 12 different wrist and hand movements of normal subjects using eight optimally placed electrodes on the forearm. Similarly, Ameri et al. (2014) used an artificial neural network (ANN), where visual training was considered better than force training to simultaneously estimate intended movements of multiple joints. Comparing the classifiers performance, Adewuyi et al. (2016) found for non-amputees and partial-hand amputees that LDA and ANN perform better than the quadratic discriminant analysis. Betthauser et al. (2018) developed a robust sparsity-based adaptive classification method to get a classification system which is appreciably less sensitive to signal deviations between training and testing. When they tested it on eight able-bodied and two transradial amputee subjects with eight electrodes pairs regularly spaced around the proximal forearm, it was found that their approach significantly outperformed other movement classification methods.

In addition to such approaches, the concept of muscle synergy was proposed to examine how the brain could efficiently command various muscles to produce different movements. For instances, to understand the posture balancing reaction of humans on a platform submitted to various perturbations in the horizontal plane, Torres-Oviedo and Ting (2007) used muscle synergies between 16 leg and lower-back human muscles. Muceli et al. (2010) found synergy among 12 muscles of the upper limb of eight subjects when reaching tasks were performed in the horizontal plane. To extract muscle synergies, various approaches can be used such as principal component analysis (PCA), independent component analysis (ICA) and non-negative matrix factorization (NMF). Amongst those, Tresch et al. (2006) considered that the NMF algorithm (Lee and Seung, 2001) was more physiologically relevant than the others given that a muscle can only be active at various contraction levels (positive) but never below rest (negative).

Features of muscle synergies are often used for classification purposes (Delis et al., 2013). For instance, Naik and Nguyen (2015) used NMF processed data to classify the finger gesture of two forearm muscles. Similarly, Rasool et al. (2016) used forearm muscles for real-time classification of hand open/close, wrist flexion/extension and forearm pronation/supination. Antuvan et al. (2016) used extreme learning machines and muscle synergy features to classify upper limb postures involved in elbow flexion/extension and shoulder flexion/protraction/retraction and rest posture. Muscle synergy has also been applied to upper limb muscles for proportional control related to prosthetic applications (Jiang et al., 2009; Ma et al., 2015).

As for our research it is focused on the multifunctional biceps brachii muscle which is involved in shoulder elevation, elbow flexion, and forearm supination (Landin et al., 2008; Jarrett et al., 2012). There is also anatomical evidence to support its multifunctionality: besides its division into two heads, its inner surface appears to be further divided into up to six compartments which are each innervated by a branch of the musculocutaneous nerve (Segal, 1992)^1^. Multifunctionality is also supported by physiological evidence: ter Haar Romeny et al. (1984) found that during different functional tasks of the upper limb, motor units of the biceps were activated at different locations within the muscle, probably due to activity in different compartments. These individual compartments can then be considered as muscles within a muscle working together to accomplish functional roles. This situation is somewhat similar to the one where anatomically different muscles work synergistically together (Bizzi and Cheung, 2013).

This paper reports on an experimental study where the biceps EMG signals are the only ones used to identify a static arm posture, out of five or eight. The study examines how successive postures could be used to develop a trajectory so as to reach a specified target in a virtual environment.

## Materials and Methods

The study was approved by the ethical committee of the Faculty of Medicine at the Université de Montréal and the 12 subjects signed a written informed consent form in accordance with the Declaration of Helsinki. To participate to the project, the inclusion criteria for each subject were: to be without any known history of neuromuscular disorders, be right-handed and aged between 20 and 35 years old; additional personal information is presented in Table 1.

**Table 1:** Information on our 12 subjects with their body mass index (BMI) and mid-upper arm circumference (MUAC).

**Subject ID**	**Height (cm)**	**Weight (kg)**	**BMI**	**MUAC (cm)**
S1	169	50	17.5	23
S2	163	48	18.1	22
S3	167	55	19.7	24
S4	157	52	21.1	25
S5	160	58	22.7	24
S6	172	80	27.0	29
S7	183	70	20.9	28
S8	180	75	23.1	30
S9	170	72	24.9	28
S10	173	77	25.7	28
S11	179	83	25.9	32
S12	183	112	33.4	37
MEAN	171.3	69.3	23.3	27.5
±SD	8.7	18.3	4.4	4.3

**Figure 1:**
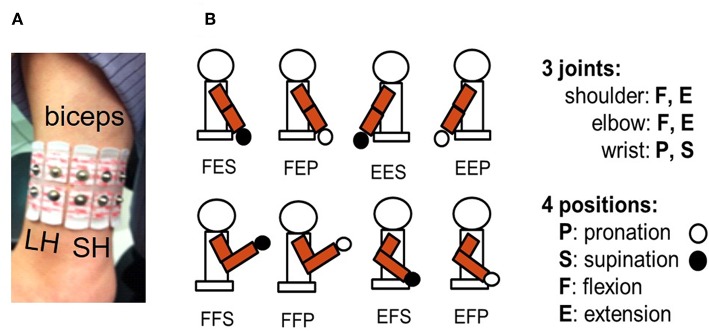
**(A)** Five bipolar surface electrodes were placed across the biceps brachii: two over the long head (LH) and three across the short head (SH). **(B)** Eight different static postures of the right upper arm were used. They are identified with a 3-tuple system [^*^,^*^,^*^] respectively representing shoulder, elbow, and wrist joints. For the first ^*^ it can be F (flexion) or E (extension) for shoulder joint posture, second ^*^ can be F or E for elbow joint posture, and third ^*^ can be P (pronation) or S (supination) for wrist joint posture.

For each subject, the borders of the biceps brachii were identified by palpation and the mid-point considered separating the short head (SH) from the long head (LH). As shown in Figure 1A, three pairs of surface electrodes were placed across the SH and two pairs across the LH while a reference electrode was placed over the acromion. To avoid the muscle's innervation zone, the upper row of electrodes was positioned 10 mm below the middle of the biceps. Ag/AgCl disc electrodes of 10 mm in diameter (Kendall H69P) were used with a 2 cm vertical and horizontal distance between center to center distances. Acquisition of the five EMG signals was done with customized electronic circuits using a differential amplifier (AD8226, Analog Devices) with a gain of 200. The amplified signals were rectified with an op amp (TL084, Texas Instruments), high-pass (6.67 Hz) and low-pass (1,240 Hz) filtered. Following the low-pass filter, a second gain of 10 was obtained using the same TL084 op amp. Following this analog processing, signals were digitized (2,000 Hz, 12 bits) with a microcontroller (ROBOTIS OpenCM9.04). On an ARM Cortex-M3 processor (72 MHz clock), a root mean squared (RMS) function was implemented with a window width of 250 ms and a large 70 ms step size due to the communication rate of 15 Hz between the microcontroller and a laptop which hosted the MATLAB software that provided data processing and a graphical user interface (GUI) for interaction with the subject.

**Figure 2:**
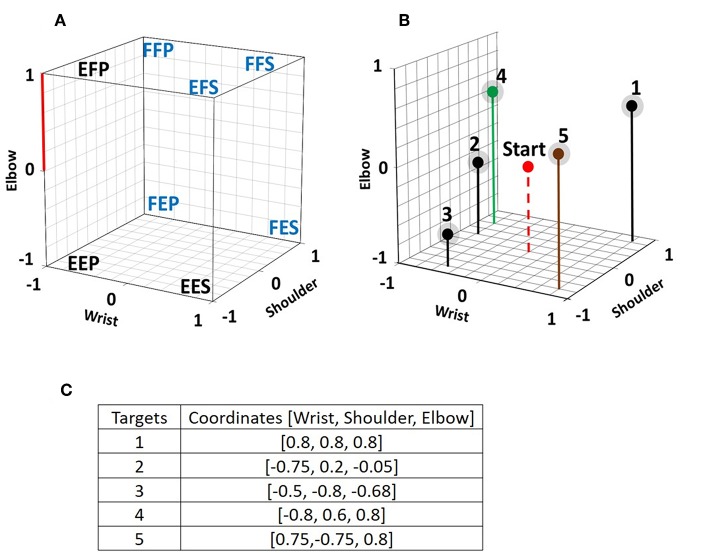
**(A)** Each of the eight chosen static postures is assigned to a corner of a virtual cube displayed on a computer screen. Each axis of the cube represents the normalized angular changes at the shoulder, the elbow, and the wrist joint. Experiments were done with the eight illustrated postures and with a subset of five postures which are identified with a blue font. On the Elbow axis, the distance between 0 and 1 is used as the reference length to which distance, length and diameter measures are compared. **(B)** 3D view of the 5 targets within the cube. In each trial, the initial position of the red sphere is in the center of the cube and subjects have to move it so that it reaches one of the targets, which have a diameter of 0.2 including their grayed surrounding. The red sphere is only a point in the program, but it is displayed with a given diameter to make it visible to the subjects. **(C)** 3D coordinates of the five targets.

While seated, subjects had to take one of eight different static postures (Figure 1B) while facing a computer screen where a cube was displayed. As shown in Figure 2A, each corner of the cube was assigned to one of the eight static postures (SP). The normalized axes of the cube were defined so the [−1 to 1] range represented the full excursion of the shoulder and elbow joints which were either extended or flexed and to the wrist joint which had the hand set either in pronation or in supination. Each intermediate position in the three axes is interpolated linearly. The distance measured between 0 and 1 on the vertical elbow axis is used as the unit against which each trajectory length and distance is measured within the cube. Before each trial (Figure 2B) the red sphere was positioned at the center of the cube and it has to be moved toward one of the targets and touch its grayish sphere (diameter: 0.2) within 120 s to be a success otherwise the trial is a failure. The coordinates of each target are shown in Figure 2C. Each subject made three trials to reach a target.

**Figure 3:**
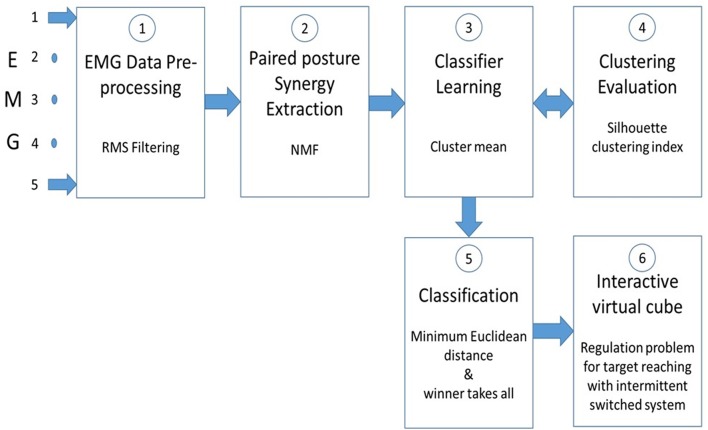
Five raw EMG signals from the biceps are smoothed ① with a RMS filter. ② sEMG signals from paired postures are concatenated before extracting muscle synergy with a non-negative matrix factorization (NMF) program. ③ Cluster mean values of the muscle synergy from pairwise postures are used to train a classifier. ④ To improve the classification accuracy, the clusters formation is evaluated with the Silhouette clustering index. ⑤ Classification of paired postures is obtained using the minimum Euclidean distance between them. In stage ⑤ a “winner takes all” method is used to make simultaneous posture classifications. In ⑥ , the virtual cube, a switched system is used to move the red sphere toward a target within the cube.

Figure 3 presents a flow chart of the MATLAB program used from EMG pre-processing up to displaying the position of the red sphere within the virtual cube. An NMF algorithm was used to extract muscle synergies from pairwise postures as done previously (He and Mathieu, 2018); details of the method are presented in Appendix 1, 2. The muscle synergy is extracted from concatenated EMG signals of two different postures and since no labeling information of the data is required, when a muscle synergy is extracted from concatenated EMG, the classifier should have the power to detect a difference between each paired posture. This power is determined by a signal-to-noise ratio (SNR) where the signal is the difference between paired postures and noise is the dissimilarity of the clusters associated to each posture in the pair. The silhouette index of Rousseeuw (1987) was used to measure the discrimination power of the muscle synergy (i.e., how easy to identify different clusters). Some details of the silhouette index are also presented in Appendix 1. In the absence of a unique solution, the NMF algorithm was applied many times (*n* = 30) on the same pairwise posture of EMG data to find the best solution, as shown in Appendix 2.

**Figure 4:**
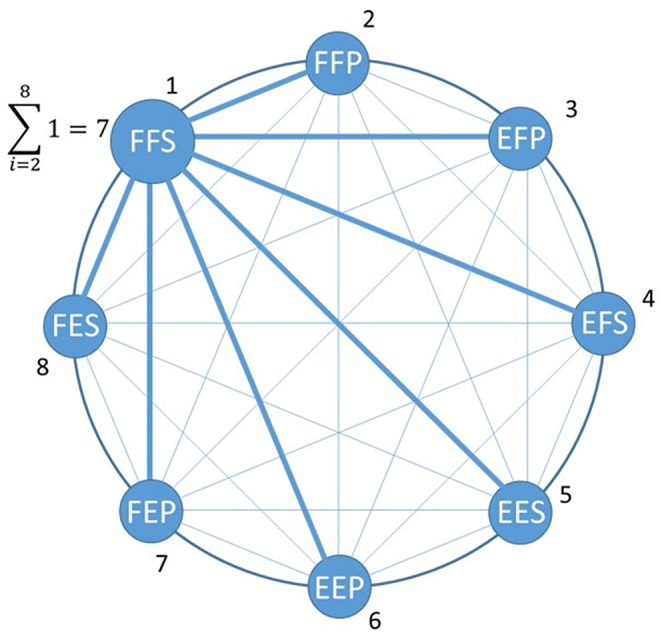
Posture classification example where the maximum number of paired posture classification is 8^*^(8–1)/2 = 28. Out of those there are 21 thin blue lines representing paired posture classifier associated with postures other than FFS which were taken by a subject. Associated with FFS are seven paired classifiers (large blue lines) which make correct classification with FFS.

For an online classification of the eight SPs of the upper limb, binary classifiers (Fürnkranz, 2002) were used with a round robin method (Park and Fürnkranz, 2007) which transforms binary classifiers into a posture classifier. The number of postures to be classified is a parameter of the classifier which determines the number of binary classifiers. With eight static postures, the number of pairwise posture classifiers needed to obtain a posture classification is 8 × (8–1)/2 = 28. The governing equation in the binary classifier is the measured Euclidean distance between the tested pattern and the learned class reference (equations A1.3, A1.4 in Appendix 1). In Figure 4, the 21 thin lines connecting a pair of eight postures represent a trained binary classifier and the seven thick lines related to the round robin method are used to identify a posture such as FFS. For the five SP condition (a subset of the eight SPs), 10 pairwise posture classifiers are used. The posture identified with the posture classifier is fed to an intermittent controller.

### Intermittent Controller

**Figure 5:**
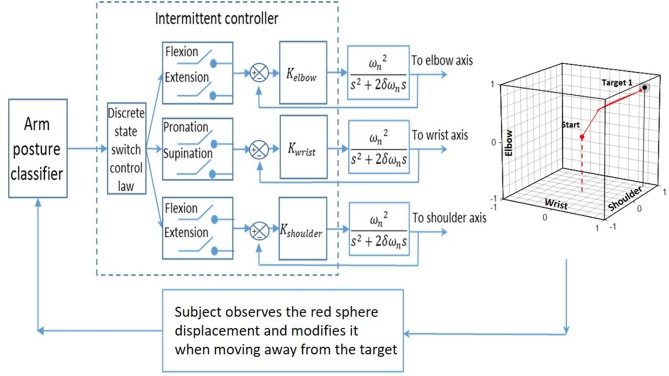
The input of an arm posture classifier is fed to an intermittent controller that determines which switch to close in order to move the red sphere accordingly with the subject's posture. The movement of the red sphere in each of the three joint axes is produced with a 2nd order system whose transfer function is given by Equation 1. The subject used the movement of the red sphere within the cube as visual feedback to produce sequential posture changes, and the objective was to reach a target in the cube.

For each trial, the initial location of the red sphere is at the center of the cube and when a first static posture taken by the subject to reach a target is identified by the classifier (Figure 5), the intermittent controller (Gawthrop et al., 2011) moves the red sphere toward the corner of the cube associated to the detected posture. If the target is not reached, additional posture changes are produced up to when the target is reached or when a 120 s time limit expires. Within the intermittent controller a discrete state switch control is used to compare the new joint posture with the previous one. Then, the activated joints are only those where a change had occurred. For example, in a FFS to FEP posture change, the shoulder joint (first F in both postures) will be inactivated and the red sphere will move, from flexion (F) to extension (E) along the elbow axis, and simultaneously on the wrist axis, from supination (S) to the pronation (P). When a change occurs simultaneously at the three joints as from EFS to FEP, the shoulder joint will only be activated and the red sphere will move, along the shoulder axis from extension (E) to flexion (F). As for the two other joints, they will remain inactive until the subject makes another posture change which does not involve the shoulder joint.

### Red Sphere Displacement

The position of the red sphere in the cube corresponds to the position of the three joints of the upper limb of a subject and a static posture change is associated with a step function input to a second-order system and the output is a continuous displacement of the red sphere toward the appropriate corner. For this second-order system (Equation 1), the resistance to the movement of the red sphere is associated with ω_*n*_ (a large value produces a larger resisting force to the displacement) and resistance to speed change of the red sphere is associated with δ (a larger value produces a larger resisting force). The transfer function of the second-order system is:

(1) $$\frac{C(s)}{R(s)}=\;\frac{{\omega_{n}}^{2}}{s^{2}+2\delta \omega_{n}s+{\omega_{n}}^{2}}$$

where *C*(*s*) is the output to the red sphere and *R*(*s*) is a step function associated with each static posture change made by the subject. The dynamic parameters of the red sphere's movement (ω_*n*_ = 0.39 and δ = 1) were the same for each subject. When a new posture is identified while the red sphere is still moving; the red sphere immediately changed its direction under the actuation of the intermittent controller. To move the red sphere anywhere within the cube, at least four static postures are needed.

### Protocol

The day before the experiment, each subject came to the laboratory to view a short video demonstrating how to produce each of the eight static postures illustrated in Figure 1B and during ~150 min, they practiced controlling the movements of the red sphere. The next day, during the experiment, targets 1, 2, 3, 4 had to be reached in succession with five SPs and targets 1, 2, 3, 5 with eight SPs. Targets 1, 2, 3 were thus considered with both the five and eight SP groups for comparison purposes. Performance of target reaching was measured with four measures: (1) time in seconds to reach a target; (2) trajectory length made by the red sphere from its initial position up to reaching a target, or in a failed trial, up to its end position when 120 s was reached; (3) number of posture changes taken to reach a target or to reach the 120 s for a failed trial; (4) the distance between the red sphere and the target when 120 s was reached for a failed trial. The Runge-Kutta 4 (RK4) method is used to obtain the trajectory length of the red sphere, which is obtained from the cumulative sum of Euclidean distances along the numerical solutions.

During the classifier training and evaluation, subjects kept their arm in each of the eight static postures for 10 s, from which the muscular synergies were extracted for classifier training. Next, they kept three times each of the five or eight static posture for 5 s, from which the obtained synergies were compared online with the trained classifier. For each trial, 30 iterations were produced by the classifier and a percentage of good classification was obtained and a mean value obtained for each subject for the five and eight SPs condition.

The NASA task load index (TLX) survey form (Appendix 3) was filled by each subject after the experiment. This is a self-evaluation of six items: mental, physical, temporal demand, and level of effort, frustration, and performance during the experiment. That feedback could provide valuable information to improve the experimental protocol and software programs used to process the information.

### Statistics

A paired *t*-test was used to compare mean results obtained by the 12 subjects when five or eight SPs were used. The tested hypothesis was that with five SPs, the mean classification results and the number of targets reached would be better than with eight SPs because remembering how to reproduce with some fidelity eight different postures is more mentally demanding than for only five. Where numbers of subjects were different for targets or postures (**Figures 7**, **8**), independent sample *t*-test were used to test the difference between the compared results. A difference was considered significant when *p* < 0.05 and the IBM SPSS Statistics software was used.

## Results

Classification % obtained during the training with five and eight SPs are shown in Table 2A. With five SPs, the classification of four subjects was very good (>90%), although it was quite poor for S12 (31%). With eight SPs, the mean classification value (of 72 ± 20%) was significantly lower (*p* = 0.001) than with five SPs (82 ± 19 %). In Table 2B, each subject's ratio of the number of reached targets out of 12 (3 trials × 4 targets) is presented. It can be observed that a good classification % in the training session was not always associated with a large ratio of reached targets. For instance, S2 and S8, who were among the five subjects with high classification performance, did not reach a single target in the eight SP condition. As expected however, S12 with the lowest classification results could only reach one target with five SPs and 0 with eight SPs (ratio: 0.08 and 0.00). For the group, the mean ratio of target reached was higher with five SPs (0.29 ± 0.18) than with eight SPs (0.24 ± 0.23) but this difference was not significant (*p* = 0.281).

**Table 2:** **(A)** In the training session, mean (± SD) classifier accuracy (%) for the 5 and 8 static postures (SP) conditions.

**Subjects**	**5 SP**	**8 SP**	**Subjects**	**5 SP**	**8 SP**
	**Mean ± SD**	**Mean ± SD**		**Ratio**	**Ratio**
**A**			**B**		
S5	99 ± 2	87 ± 20	S3	0.67	0.75
S11	95 ± 4	68 ± 35	S8	0.50	0.00
S1	94 ± 6	88 ± 25	S7	0.50	0.25
S2	90 ± 10	83 ± 20	S1	0.42	0.58
S8	89 ± 9	79 ± 21	S6	0.33	0.33
S3	87 ± 14	75 ± 43	S5	0.25	0.25
S4	87 ± 21	90 ± 11	S9	0.25	0.25
S7	87 ± 15	85 ± 13	S11	0.17	0.17
S9	85 ± 15	72 ± 26	S4	0.17	0.17
S10	76 ± 16	64 ± 26	S2	0.08	0.00
S6	60 ± 36	49 ± 45	S10	0.08	0.08
S12	31 ± 45	22 ± 42	S12	0.08	0.00
Mean ± SD	82 ± 19	72 ± 20	Mean ± SD	0.29 ± 0.18	0.24 ± 0.23

*Mean values of each subject are obtained from three trials of 5 s in each of the five or in each of the eight static postures. The subjects are sorted from highest to lowest performance in the 5 SP condition. The difference between 5 and 8 SP results is significant (p <0.05). **(B)** Success ratio [number of successful trials over 12 trials (4 targets × 3 times)] by each subject. The subjects are sorted from highest to lowers performance in the 5 SP condition. S3 was the best performer. Between the 5 and 8 SP results the paired t-test value is 0.28*.

**Figure 6:**
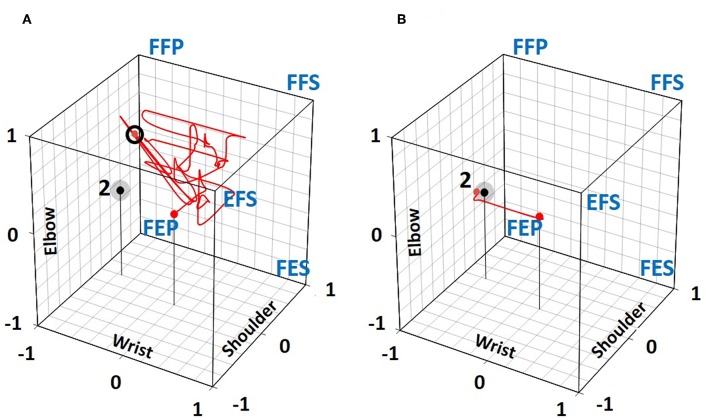
Trajectories taken by S3 to move the red sphere toward target 2 in the 5 SP condition. **(A)** An unsuccessful 1st trial in spite of 72 posture changes where the final red sphere position after 120 s was at a distance of 0.68 (small black circle) from the target. **(B)** In the 3rd trial, the target was reached within 9 s with only 4 posture changes.

Figure 6 illustrates two trajectories of the red sphere which was controlled by S3 trying to reach target two with five SPs. In Figure 6A, an example of a failed trial is shown, where in spite of 72 posture changes made during 120 s, the red sphere was still at a distance of 0.68^2^ from target two after a trajectory length of 14.7^2^. The same subject was far more successful in another trial (Figure 6B) where the same target was reached with only four posture changes within 9 s.

**Figure 7:**
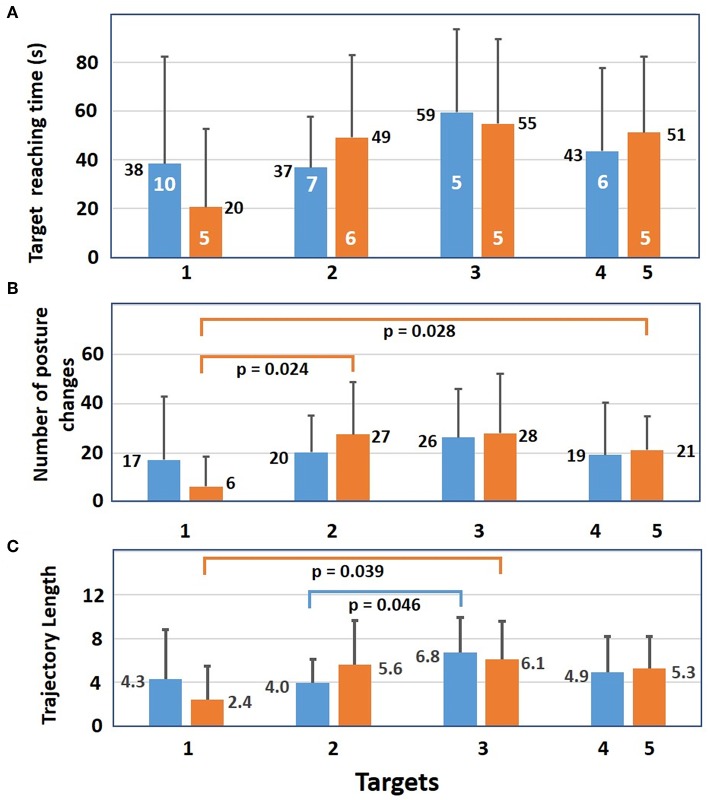
For successful trials, mean results (+sd) of the 12 subjects with 5 (blue bars) and with 8 static postures (orange bars). **(A)** Mean time needed to reach each target. Number of subjects who reached a target at least once is shown in white. **(B)** Mean number of posture changes needed to reach each target. For 8 static postures, there is a significant difference in the number of posture changes between targets 1 and 2 as well as between target 1 and 5. **(C)** Mean trajectory length needed to reach each target. Differences between targets 1 and 3 are significant for 8 static postures as well as targets 2 and 3 for five postures. Significance of independent samples *t*-test level is *p* ≤ 0.05.

The mean time to successfully reach the targets is shown in Figure 7A. Target one was the easiest to reach with a mean time of 38 and 20 s for the five SP and eight SP condition, respectively, and target three was the most difficult to reach with 59 and 55 s. These mean values are the results of significant variations among the subjects and no significant difference was found between those results. Reaching target one was achieved with a smaller number of posture changes (Figure 7B) than for the other targets. With eight SPs, a significant difference was found between targets one and two and between one and five. For trajectory length (Figure 7C), a significant difference was obtained with eight SPs between targets one and three and with five SPs between targets two and three.

**Figure 8:**
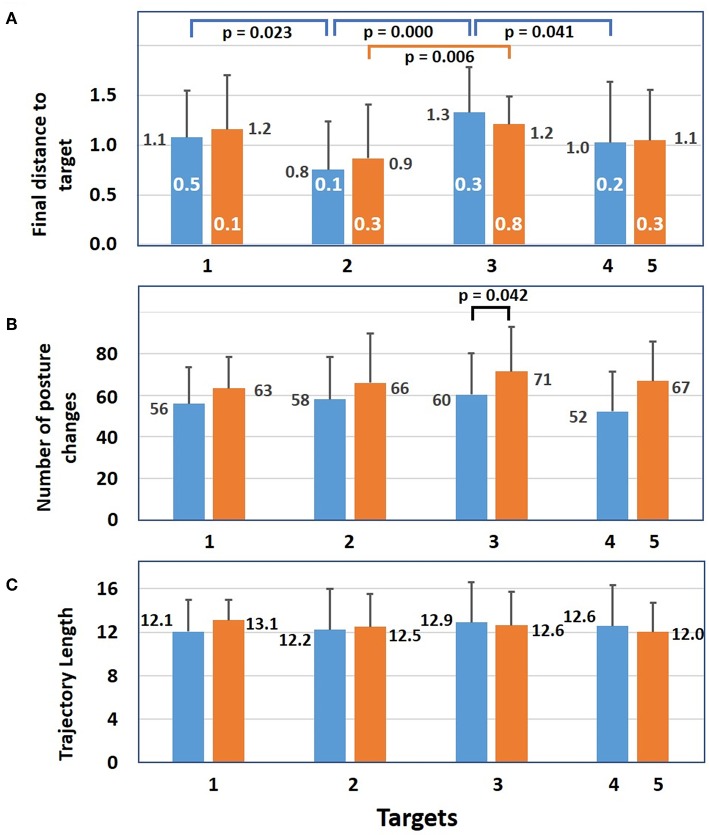
Results of failed trials. **(A)** Mean distance (+sd) between the red sphere and a target at 120 s for 5 (blue bars) and for eight static postures (orange bars). White numbers represent the minimum distance between the red sphere and a target at 120 s. For the 5 SP condition, differences between targets 1 and 2, targets 2 and 3, and targets 3 and 4 are significant. For the 8 SP condition, the difference between targets 2 and 3 is significant. **(B)** Mean number of posture changes at the end of each trial. For target 3 the difference between 5 and 8 SP conditions is significant. **(C)** For each target, the mean trajectory length of the red sphere during 120 s (independent samples *t*-test significance is *p* ≤ 0.05).

Results for failed trials are shown in Figure 8. In Figure 8A, it is seen that mean distances separating the red sphere from target two at the end of 120 s are smaller than those for the other targets. For the five SP condition the differences are significant between targets 1 2, 3, and 4 while for the eight SP condition the difference is only significant between targets two and three. While mean distance varied between 0.8 and 1.3, the smallest distance (0.1) was found with five SPs for target two and with eight SPs for target one and the largest was 0.8 (white number) for the eight SPs condition at target three. As for the number of mean posture changes (Figure 8B), they were always smaller with five SPs than with eight SPs and the only significant difference was for five and eight SPs at target three. The mean trajectory lengths of the red sphere (Figure 8C) were all equally elevated for five and eight SPs.

The results of the NASA task load form are presented in Table 3. As a group, subjects considered that physical and mental demands to identify which static posture to choose to move the red sphere were high (15.3/20 and 14.0/20). As for the temporal demand, the limit of 120 s appeared adequate and the time spend in the lab not too long (10.4/20). In general, subjects were not very satisfied with their performance (7.2 ± 3.7) and somewhat frustrated (12.8 ± 4.9) from not having reached the targets more often. At the individual level, the best performer (S3) ranked both the physical and mental demands at a high 17/20 and frustration at the highest score of 20/20, for not being able to reproduce the postures correctly in order to reach more targets. While S2 got over 80% for the training classifier accuracy results (Table 2A), this subject could only reach one target (Table 2B). This subject considered the performance quite low (4/20) and was very frustrated (16/20). While the other low performer (S12) missed most of the targets, this subject considered, unexpectedly, that the experiment was not very mentally demanding and was not frustrating.

**Table 3:** NASA task load index **(TLX)** rates six factors (left column of the table) that are used to assess the subjective workload associated with the experiment.

**TLX**	**Mean ± SD (*n* = 12)**	**Best performer (S3)**	**Lowest performer 1 (S2)**	**Lowest performer 2 (S12)**
Physical demand	15.3 ± 2.8	17	14	13
Mental demand	14.0 ± 3.6	17	15	9
Temporal demand	10.4 ± 6.0	11	14	13
Own performance	7.2 ± 3.7	15	4	5
Own frustration	12.8 ± 4.9	20	16	8
Own effort	12.4 ± 3.4	15	13	11

*The three top factors are related to constraints associated with the tasks to be realized (demand) and the three bottom factors are related to the feelings associated with those tasks (own). Each factor is scaled from 0 to 20: higher values represents higher demand or higher performance. Mean values of the 12 subjects are shown in the second column of the table. Individual results are shown for the best performer and for the two lowest performing subjects*.

## Discussion

Modern upper limb myoelectric prostheses are now able to produce many different movements. However, following an amputation, the number of available muscles to control them is reduced and strategies to alleviate that shortage have to be developed. When the biceps brachii is still functional, one strategy could be the extraction of more than one control signal from that muscle. To investigate that possibility with non-amputee subjects, five pairs of surface electrodes were put across this multifunctional muscle. With two postures for each shoulder, elbow and wrist joints eight different static postures in the sagittal plane were used to control the displacement of a cursor toward different targets placed within a virtual cube. Results were obtained in two experimental conditions: one with five out of the eight static postures and one with all eight static postures.

To associate a red sphere direction to each of the eight static postures, a training phase was used. After having done that with each subject, we verified the ability of a classifier to correctly recognize each of the eight SPs. As shown in Table 2A, an important difference is observed between results of S5 at the top of the table and S12 at its bottom. Ability to remember how to reproduce with high fidelity five or eight different static arm postures was thus quite variable among our subjects. With, a group mean value with five SPs being significantly larger than with eight SPs (*p* = 0.001), this confirms that remembering how to duplicate five postures is significantly easier than duplicating eight SPs.

Since the classifier accuracy of the above results were obtained online, no data was available for an offline cross-validation. However, the classifier training was with unsupervised synergies and their features formed non-overlapping clusters. Thus, the discrimination capacity of the learned linear classifier is not an important concern since the receiver operating characteristic curve (ROC) of those features always occupies the upper half triangle. However, it is still possible that when a subject chooses a static posture, a misclassification occurs, causing the cursor to move in an unexpected direction, which is confusing for the subject. To prevent such situations, the use of a sparse representation of the classified postures as proposed by Betthauser et al. (2018) has to be added to our programs.

From the starting position of the red sphere (Figure 2B), targets one and five (which are located near a corner of the cube) could easily be reached by taking the FFS or EFS posture, respectively because the initial posture that starts the movement of the red sphere actives movement in all three joint axes toward the corner corresponding to that posture. Those easy reaching strategies were used by only few subjects. Targets located at a distance from a corner were more difficult to reach since different postures had to be sequentially taken to reach them.

Among the eight SPs, different subsets of five postures could have been chosen. The present choice was based on the main contribution of the biceps to the elbow flexion and forearm supination. As for the low mean target reaching ratios (<0.30, Table 2B) and especially for subjects S2 and S12 who reached only one target over 12 trials with five SPs and 0 targets with eight SPs, classification accuracy could be improved by replacing the present classifier by a support vector machine or an artificial neural network classifier. Also, the short training period the day before the experiment could be replaced by more training sessions as illustrated by one person of the lab who, having repeated the protocol four times, reached a 90% success rate with five SPs and 58% with eight SPs (unpublished results).

Classification results could also be improved by the addition of anatomical information on the biceps when the upper limb is in different postures. With an ultrasound probe placed at the biceps level where recording electrodes had been previously placed, changes in its shape and displacements relative to the skin surface were observed (unpublished results). In the future, with ultrasound images obtained before an experiment, position of the electrodes over the biceps could be optimized.

From the results of the NASA task load index, physical and mental demands have the highest scores indicating that our present approach to reach targets is not very intuitive. The difficulty of controlling their prostheses frequently leads amputees to leave them in a closet. Thus, it is suggested to replace the step by step cursor control of a red sphere in a virtual cube by a more realistic situation where the biceps' synergy would control a small humanoid robot for reaching objects within an arm's length. This would be a more realistic situation to the one shown in a video where the experimented person of the lab controlled a small humanoid robot arm with muscular synergies of the biceps (http://www.igb.umontreal.ca/).

## Conclusion

We present a proof of concept that the muscular synergy extracted from a single muscle, the biceps brachii, could facilitate the control of an upper limb prosthesis. This was demonstrated by collecting five surface EMG signals of the biceps of 12 normal subjects who put their arm in eight different static postures. Using a non-negative matrix factorization program, three muscular synergies were extracted and following a training session, a classifier could identify each of those eight postures. Then, within a virtual cube displayed on a screen, subjects could, with five and eight different static postures, move a red sphere toward the targets. The number of targets reached was higher with five choices of posture than with eight choices. The reasons for a low mean number of reached targets (around 30%) were a lack of training of our subjects before the experiment and a classifier that was not lenient enough. While the biceps may not be available for above elbow amputees, a muscular synergy may then be extracted from muscles near the shoulder such as the deltoid, the pectoralis major and the latissimus dorsi, which are also multifunctional.

## Data Availability Statement

The datasets generated for this study are available on request to the corresponding author.

## Ethics Statement

The studies involving human participants were reviewed and approved by Comité d'éthique de la recherche en santé, Université de Montréal. The patients/participants provided their written informed consent to participate in this study.

## Author Contributions

LH developed the interactive control system for target reaching, conducted the data acquisition, compiled the results, and wrote the initial draft of the article. PM designed the evaluation protocol, supervised the study, revised the article, and obtained the funding. Each author approved the final version of the manuscript.

### Conflict of Interest

The authors declare that the research was conducted in the absence of any commercial or financial relationships that could be construed as a potential conflict of interest.

## Footnotes

^1^Anatomically, compartments are not unique to the biceps brachii. They are also present in the deltoid, the pectoralis major and the latissimus dorsi at the shoulder level (Brown et al., 2007) which are less frequently studied than the biceps.

^2^Length is referenced to the 0 to1 distance on the elbow axis (Figure 2A).
