# Supplementary material for: Biceps Brachii Muscle Synergy and Target Reaching in a Virtual Environment
Source: Front Neurorobot. 2019 Dec 10;13:100. doi: 10.3389/fnbot.2019.00100 (PMC6914832; doi:10.3389/fnbot.2019.00100)
Supplement: Supplementary file 1 [file Presentation_1.zip › Appendix.docx]

# Appendix 1

## Synergy extraction from pairwise postures

For muscle synergy extraction, EMG signals from concatenated pairwise postures are used. The process of muscle synergy extraction involves a dimensionality reduction where three muscle synergies are extracted from 5 EMGs of the biceps. Given a concatenated EMG dataset, E, two matrices H and W are obtained with the NMF algorithm

$E^{5\times n}=W^{5\times3}\cdot H^{3\times n}$ (A1.1)

where $W^{5\times3}$ is the muscle synergy matrix, and $H^{3\times n}$ is the matrix of muscle synergy coefficients. Given pairwise postures, the synergy coefficient from the first posture is defined as $H_{1}$ and the second is $H_{2}$. Their mean values i.e. $\bar{H}_{1}$ and $\bar{H}_{2}$ are used to train the minimum distance classifier. The muscle synergy matrix $W$ is used for estimation of the synergy coefficient from the EMG signals acquired during evaluation as follows:

${\hat{H}_{i}}^{3\times1}= {W^{5\times3}}^{-1}{E_{i}}^{5\times1}$ (A1.2)

where $i$ is the sample number in sequentially acquired EMG signals. The distance measured from the estimated synergy coefficient is given as follows

$D_{1}^{i}= \left| \hat{H}_{i}- \bar{H}_{1} \right|$ (A1.3)

$D_{2}^{i}= \left| \hat{H}_{i}- \bar{H}_{2} \right|$ (A1.4)

where the Euclidean distance is used as the metric measure. The minimum distance classifier determines the classification result as follows

$$if D_{1}^{i} < D_{2}^{i}, then the posture associated with \hat{H}_{i} is of 1^{st} class, otherwise 2^{nd}class$$

The classification presented here is called the pairwise posture classifier and it uses the muscle synergy coefficient obtained from the paired postures for classifier training and estimation of the muscle synergy from EMG using the pseudo inverse of the synergy matrix, $W^{5\times3}$.

## Silhouette index (Rousseeuw, 1987)

To improve the discrimination power of the metric based classifier, the Silhouette clustering validation index, $S$, is used to evaluate the muscle synergy clustering from the pairwise postures. The Silhouette index is given as follows:

$S_{i}= \frac{b_{i}- a_{i}}{\max(a_{i},b_{i})}$ (A1.5)

where $a_{i}$ is the average distance of synergy coefficient $i$ in one cluster to all other synergy coefficient values in the same cluster, and $b_{i}$ is the minimum average distance from synergy coefficient $i$ in one cluster to all synergy coefficients in the other cluster. The value of $S_{i}$ varies from $-1$ to $1$. A large $S_{i}$ value means the discrimination power is high. The average value of $S_{i}$ is used to determine the discrimination power of muscle synergy coefficients obtained from pairwise postures:

$S= \frac{1}{n}\sum_{i=1}^{n} S_{i}$ (A1.6)

# Appendix 2

## Multiple synergy extraction

In Algorithm 1 (below), $T$ is the predefined number of reinitiated NMF extractions, $S$ is the Silhouette index value (initialized at -1), $E$ is the concatenated $\mathrm{EMG}$ signal from paired postures, $L$ is the cluster label, and $W$ and $H$ are the synergy matrix and synergy coefficients from the $\mathrm{NMF}$ algorithm.

**Algorithm 1** Clustering Validation Index for Paired Postures (*Pos_1_*, *Pos_2_*)

1: **procedure** CLUSER VALIDATION (*T, S, E, L, W, H*)

2: *T* = 30

3: *S* = -1

4: {*E*, *L*} = {*Pos_1_*, *Pos_2_*}

5: {*W*, *H*} = {***I***, ***0***}

6: **for** each *i* in *T* **do**

7: {new*W*, new*H*} = NMF(*E*)

8: new*S* = Silhouette Index (new*H*, *L*)

9: **if** new*S* > *S* **then**

10: *S* = new*S*

11: {*W*, *H*} = {new*W*, new*H*}

12: **end if**

13: **end for**

14: **Return** *S*, *W*, *H*, *L*

15: **end procedure**

# Appendix 3

**
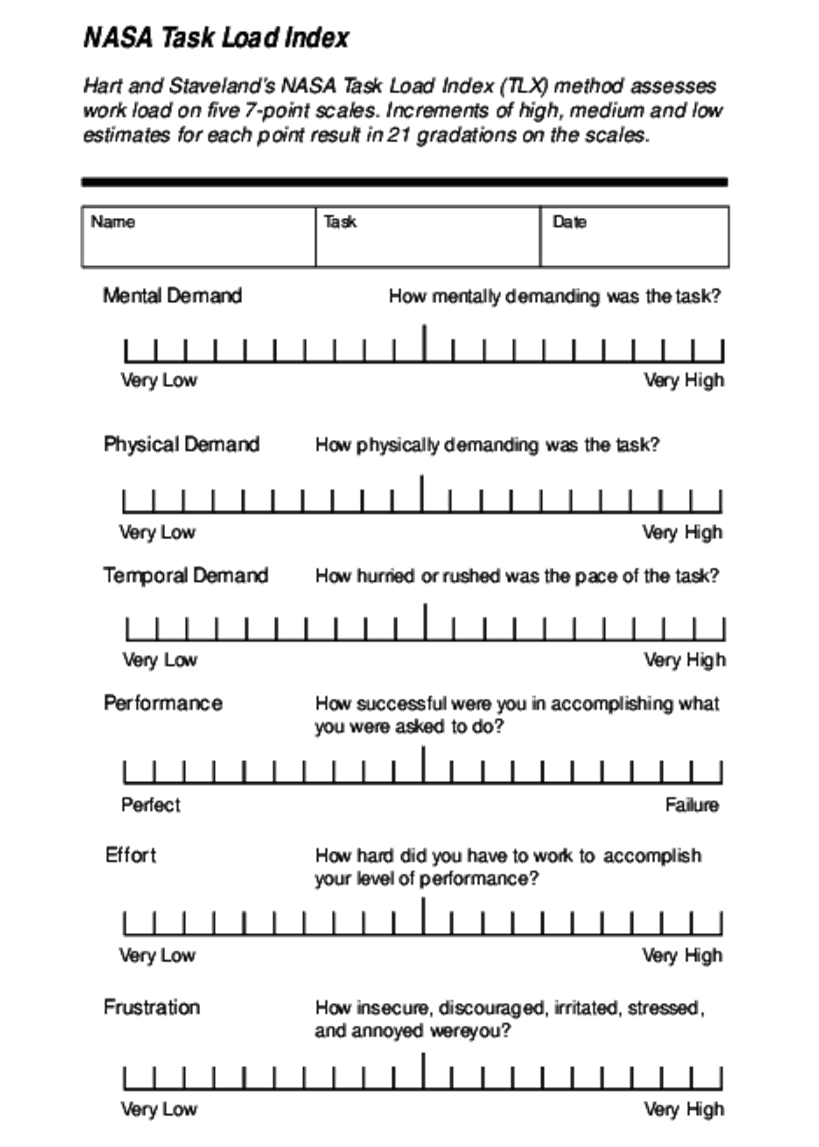
**
